# Bone regeneration: current concepts and future directions

**DOI:** 10.1186/1741-7015-9-66

**Published:** 2011-05-31

**Authors:** Rozalia Dimitriou, Elena Jones, Dennis McGonagle, Peter V Giannoudis

**Affiliations:** 1 Department of Trauma and Orthopaedics, Academic Unit, Clarendon Wing, Leeds Teaching Hospitals NHS Trust, Great George Street, Leeds LS1 3EX, UK; 2 UK Leeds NIHR Biomedical Research Unit, Leeds Institute of Molecular Medicine, Beckett Street, Leeds, LS9 7TF, UK; 3 Section of Musculoskeletal Disease, Leeds Institute of Molecular Medicine, University of Leeds and Chapel Allerton Hospital, Chapeltown Road, Leeds, UK

## Abstract

Bone regeneration is a complex, well-orchestrated physiological process of bone formation, which can be seen during normal fracture healing, and is involved in continuous remodelling throughout adult life. However, there are complex clinical conditions in which bone regeneration is required in large quantity, such as for skeletal reconstruction of large bone defects created by trauma, infection, tumour resection and skeletal abnormalities, or cases in which the regenerative process is compromised, including avascular necrosis, atrophic non-unions and osteoporosis. Currently, there is a plethora of different strategies to augment the impaired or 'insufficient' bone-regeneration process, including the 'gold standard' autologous bone graft, free fibula vascularised graft, allograft implantation, and use of growth factors, osteoconductive scaffolds, osteoprogenitor cells and distraction osteogenesis. Improved 'local' strategies in terms of tissue engineering and gene therapy, or even 'systemic' enhancement of bone repair, are under intense investigation, in an effort to overcome the limitations of the current methods, to produce bone-graft substitutes with biomechanical properties that are as identical to normal bone as possible, to accelerate the overall regeneration process, or even to address systemic conditions, such as skeletal disorders and osteoporosis.

## Introduction

Bone possesses the intrinsic capacity for regeneration as part of the repair process in response to injury, as well as during skeletal development or continuous remodelling throughout adult life [1,2]. Bone regeneration is comprised of a well-orchestrated series of biological events of bone induction and conduction, involving a number of cell types and intracellular and extracellular molecular-signalling pathways, with a definable temporal and spatial sequence, in an effort to optimise skeletal repair and restore skeletal function [2,3]. In the clinical setting, the most common form of bone regeneration is fracture healing, during which the pathway of normal fetal skeletogenesis, including intramembranous and endochondral ossification, is recapitulated [4]. Unlike in other tissues, the majority of bony injuries (fractures) heal without the formation of scar tissue, and bone is regenerated with its pre-existing properties largely restored, and with the newly formed bone being eventually indistinguishable from the adjacent uninjured bone [2]. However, there are cases of fracture healing in which bone regeneration is impaired, with, for example, up to 13% of fractures occurring in the tibia being associated with delayed union or fracture non-union [5]. In addition, there are other conditions in orthopaedic surgery and in oral and maxillofacial surgery in which bone regeneration is required in large quantity (beyond the normal potential for self-healing), such as for skeletal reconstruction of large bone defects created by trauma, infection, tumour resection and skeletal abnormalities, or cases in which the regenerative process is compromised, including avascular necrosis and osteoporosis.

## Current clinical approaches to enhance bone regeneration

For all the aforementioned cases in which the normal process of bone regeneration is either impaired or simply insufficient, there are currently a number of treatment methods available in the surgeon's armamentarium, which can be used either alone or in combination for the enhancement or management of these complex clinical situations, which can often be recalcitrant to treatment, representing a medical and socioeconomic challenge. Standard approaches widely used in clinical practice to stimulate or augment bone regeneration include distraction osteogenesis and bone transport [6,7], and the use of a number of different bone-grafting methods, such as autologous bone grafts, allografts, and bone-graft substitutes or growth factors [8,9]. An alternative method for bone regeneration and reconstruction of long-bone defects is a two-stage procedure, known as the Masquelet technique. It is based on the concept of a "biological" membrane, which is induced after application of a cement spacer at the first stage and acts as a 'chamber' for the insertion of non-vascularised autograft at the second stage [10]. There are even non-invasive methods of biophysical stimulation, such as low-intensity pulsed ultrasound (LIPUS) and pulsed electromagnetic fields (PEMF) [11-13], which are used as adjuncts to enhance bone regeneration.

During distraction osteogenesis and bone transport, bone regeneration is induced between the gradually distracted osseous surfaces. A variety of methods are currently used to treat bone loss or limb-length discrepancies and deformities, including external fixators and the Ilizarov technique [6,7], combined unreamed intramedullary nails with external monorail distraction devices [14], or intramedullary lengthening devices [15]. However, these methods are technically demanding and have several disadvantages, including associated complications, requirement for lengthy treatment for both the distraction (1 mm per day) and the consolidation period (usually twice the distraction phase), and effects on the patient's psychology and well-being [6,7].

Bone grafting is a commonly performed surgical procedure to augment bone regeneration in a variety of orthopaedic and maxillofacial procedures, with autologous bone being considered as the 'gold standard' bone-grafting material, as it combines all properties required in a bone-graft material: osteoinduction (bone morphogenetic proteins (BMPs) and other growth factors), osteogenesis (osteoprogenitor cells) and osteoconduction (scaffold) [16]. It can also be harvested as a tricortical graft for structural support [16], or as a vascularised bone graft for restoration of large bone defects [17] or avascular necrosis [18]. A variety of sites can be used for bone-graft harvesting, with the anterior and posterior iliac crests of the pelvis being the commonly used donor sites. Recently, with the development of a new reaming system, the reamer-irrigator-aspirator (RIA), initially developed to minimise the adverse effects of reaming during nailing of long-bone fractures, the intramedullary canal of long bones has been used as an alternative harvesting site, providing a large volume of autologous bone graft [19]. Furthermore, because it is the patient's own tissue, autologous bone is histocompatible and non-immunogenic, reducing to a minimum the likelihood of immunoreactions and transmission of infections. Nevertheless, harvesting requires an additional surgical procedure, with well-documented complications and discomfort for the patient, and has the additional disadvantages of quantity restrictions and substantial costs [20-22].

An alternative is allogeneic bone grafting, obtained from human cadavers or living donors, which bypasses the problems associated with harvesting and quantity of graft material. Allogeneic bone is available in many preparations, including demineralised bone matrix (DBM), morcellised and cancellous chips, corticocancellous and cortical grafts, and osteochondral and whole-bone segments, depending on the recipient site requirements. Their biological properties vary, but overall, they possess reduced osteoinductive properties and no cellular component, because donor grafts are devitalised via irradiation or freeze-drying processing [23]. There are issues of immunogenicity and rejection reactions, possibility of infection transmission, and cost [8,23].

Bone-graft substitutes have also been developed as alternatives to autologous or allogeneic bone grafts. They consist of scaffolds made of synthetic or natural biomaterials that promote the migration, proliferation and differentiation of bone cells for bone regeneration. A wide range of biomaterials and synthetic bone substitutes are currently used as scaffolds, including collagen, hydroxyapatite (HA), β-tricalcium phosphate (β-TCP) and calcium-phosphate cements, and glass ceramics [8,23], and the research into this field is ongoing. Especially for reconstruction of large bone defects, for which there is a need for a substantial structural scaffold, an alternative to massive cortical auto- or allografts is the use of cylindrical metallic or titanium mesh cages as a scaffold combined with cancellous bone allograft, DBM or autologous bone [24,25].

## Limitations of current strategies to enhance bone regeneration

Most of the current strategies for bone regeneration exhibit relatively satisfactory results. However, there are associated drawbacks and limitations to their use and availability, and even controversial reports about their efficacy and cost-effectiveness. Furthermore, at present there are no heterologous or synthetic bone substitutes available that have superior or even the same biological or mechanical properties compared with bone. Therefore, there is a necessity to develop novel treatments as alternatives or adjuncts to the standard methods used for bone regeneration, in an effort to overcome these limitations, which has been a goal for many decades. Even back in the 1950s, Professor Sir Charnley, a pioneer British orthopaedic surgeon, stated that 'practically all classical operations of surgery have now been explored, and unless some revolutionary discovery is made which will put the control of osteogenesis in the surgeon's power, no great advance is likely to come from modification of their detail' [26].

**Figure 1:**
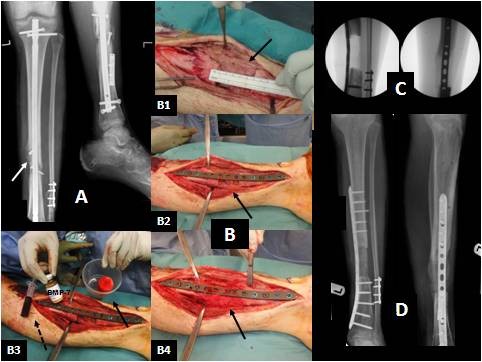
**Male patient 19 years of age with infected non-union after intramedullary nailing of an open tibial fracture**. **(A). **Anteroposterior (AP) and lateral X-rays of the tibia illustrating osteolysis (white arrow) secondary to infection. The patient underwent removal of the nail, extensive debridement and a two-staged reconstruction of the bone defect, using the induced membrane technique for bone regeneration (the Masquelet technique). **(B) **Intraoperative pictures showing: (1) a 60 mm defect of the tibia (black arrow) at the second stage of the procedure; (2) adequate mechanical stability was provided with internal fixation (locking plate) bridging the defect, while the length was maintained (black arrow); (3) maximum biological stimulation was provided using autologous bone graft harvested from the femoral canal (black arrow, right), bone-marrow mesenchymal stem cells (broken arrow, left) and the osteoinductive factor bone morphogenetic protein-7 (centre); (4) the graft was placed to fill the bone defect (black arrow). **(C) **Intraoperative fluoroscopic images showing the bone defect after fixation. **(D) **Postoperative AP and lateral X-rays at 3 months, showing the evolution of the bone regeneration process with satisfactory incorporation and mineralisation of the graft (photographs courtesy of PVG).

Since then, our understanding of bone regeneration at the cellular and molecular level has advanced enormously, and is still ongoing. New methods for studying this process, such as quantitative three-dimensional microcomputed tomography analyses, finite element modelling, and nanotechnology have been developed to further evaluate the mechanical properties of bone regenerate at the microscopic level. In addition, advances made in cellular and molecular biology have allowed detailed histological analyses, *in vitro *and *in vivo *characterisation of bone-forming cells, identification of transcriptional and translational profiles of the genes and proteins involved in the process of bone regeneration and fracture repair, and development of transgenic animals to explore the role of a number of genes expressed during bone repair, and their temporal and tissue-specific expression patterns [27]. With the ongoing research in all related fields, novel therapies have been used as adjuncts or alternatives to traditional bone-regeneration methods. Nevertheless, the basic concept for managing all clinical situations requiring bone regeneration, particularly the complex and recalcitrant cases, remains the same, and must be applied. Treatment strategies should aim to address all (or those that require enhancement) prerequisites for optimal bone healing, including osteoconductive matrices, osteoinductive factors, osteogenic cells and mechanical stability, following the 'diamond concept' suggested for fracture healing (Figure 1) [28].

## BMPs and other growth factors

With improved understanding of fracture healing and bone regeneration at the molecular level [29], a number of key molecules that regulate this complex physiological process have been identified, and are already in clinical use or under investigation to enhance bone repair.

Of these molecules, BMPs have been the most extensively studied, as they are potent osteoinductive factors. They induce the mitogenesis of mesenchymal stem cells (MSCs) and other osteoprogenitors, and their differentiation towards osteoblasts. Since the discovery of BMPs, a number of experimental and clinical trials have supported the safety and efficacy of their use as osteoinductive bone-graft substitutes for bone regeneration. With the use of recombinant DNA technology, BMP-2 and BMP-7 have been licensed for clinical use since 2002 and 2001 respectively [30]. These two molecules have been used in a variety of clinical conditions including non-union, open fractures, joint fusions, aseptic bone necrosis and critical bone defects [9]. Extensive research is ongoing to develop injectable formulations for minimally invasive application, and/or novel carriers for prolonged and targeted local delivery [31].

Other growth factors besides BMPs that have been implicated during bone regeneration, with different functions in terms of cell proliferation, chemotaxis and angiogenesis, are also being investigated or are currently being used to augment bone repair [32,33], including platelet-derived growth factor, transforming growth factor-β, insulin-like growth factor-1, vascular endothelial growth factor and fibroblast growth factor, among others [29]. These have been used either alone or in combinations in a number of *in vitro *and *in vivo *studies, with controversial results [32,33]. One current approach to enhance bone regeneration and soft-tissue healing by local application of growth factors is the use of platelet-rich plasma, a volume of the plasma fraction of autologous blood with platelet concentrations above baseline, which is rich in many of the aforementioned molecules [34].

'Orthobiologics' and the overall concept to stimulate the local 'biology' by applying growth factors (especially BMPs, because these are the most potent osteoinductive molecules) could be advantageous for bone regeneration or even for acceleration of normal bone healing to reduce the length of fracture treatment. Their clinical use, either alone or combined with bone grafts, is constantly increasing. However, there are several issues about their use, including safety (because of the supraphysiological concentrations of growth factors needed to obtain the desired osteoinductive effects), the high cost of treatment, and more importantly, the potential for ectopic bone formation [35].

Currently BMPs are also being used in bone-tissue engineering, but several issues need to be further examined, such as optimum dosage and provision of a sustained, biologically appropriate concentration at the site of bone regeneration, and the use of a 'cocktail' of other growth factors that have shown significant promising results in preclinical and early clinical investigation [32] or even the use of inhibitory molecules in an effort to mimic the endogenous 'normal' growth-factor production. Nanoparticle technology seems to be a promising approach for optimum growth-factor delivery in the future of bone-tissue engineering [36]. Nevertheless, owing to gaps in the current understanding of these factors, it has not been possible to reproduce *in vivo *bone regeneration in the laboratory.

## MSCs

An adequate supply of cells (MSCs and osteoprogenitors) is important for efficient bone regeneration. The current approach of delivering osteogenic cells directly to the regeneration site includes use of bone-marrow aspirate from the iliac crest, which also contains growth factors. It is a minimally invasive procedure to enhance bone repair, and produces satisfactory results [37]. However, the concentration and quality of MSCs may vary significantly, depending on the individual (especially in older people) [38,39], the aspiration sites and techniques used [39], and whether further concentration of the bone marrow has been performed [37], as bone-marrow aspiration concentrate (BMAC) is considered to be an effective product to augment bone grafting and support bone regeneration [40,41]. Overall, however, there are significant ongoing issues with quality control with respect to delivering the requisite number of MSCs/osteoprogenitors to effect adequate repair responses [40].

Issues of quantity and alternative sources of MSCs are being extensively investigated. Novel approaches in terms of cell harvesting, *in vitro *expansion and subsequent implantation are promising [42-44], because *in vitro *expansion can generate a large number of progenitor cells. However, such techniques add substantial cost and risks (such as contamination with bacteria or viruses), may reduce the proliferative capacity of the cells and are time-consuming requiring two-stage surgery [45]. This strategy is already applied for cartilage regeneration [46]. Alternative sources of cells, which are less invasive, such as peripheral blood [47] and mesenchymal progenitor cells from fat [48], muscle, or even traumatised muscle tissue after debridement [49], are also under extensive research. However, the utility of fat-derived MSCs for bone-regeneration applications is debatable, with some studies showing them to be inferior to bone-marrow-derived MSCs in animal models [50,51], and the evidence for a clinically relevant or meaningful population of circulating MSCs also remains very contentious [52].

It is fair to say that the role of MSCs in fracture repair is still in its infancy, largely due to a lack of studies into the biology of MSCs *in vivo *in the fracture environment. This to a large extent relates to the historical perceived rarity of '*in vivo *MSCs' and also to a lack of knowledge about *in vivo *phenotypes. The *in vivo *phenotype of bone-marrow MSCs has been recently reported [53] and, even more recently, it has been shown that this population was actually fairly abundant *in vivo *in normal and pathological bone [54]. This knowledge opens up novel approaches for the characterisation and molecular profiling of MSCs *in vivo *in the fracture environment. This could be used to ultimately improve outcomes of fracture non-union based on the biology of these key MSC reparative cells.

## Scaffolds and bone substitutes

Although they lack osteoinductive or osteogenic properties, synthetic bone substitutes and biomaterials are already widely used in clinical practice for osteoconduction. DBM and collagen are biomaterials, used mainly as bone-graft extenders, as they provide minimal structural support [8]. A large number of synthetic bone substitutes are currently available, such as HA, β-TCP and calcium-phosphate cements, and glass ceramics [8,23]. These are being used as adjuncts or alternatives to autologous bone grafts, as they promote the migration, proliferation and differentiation of bone cells for bone regeneration. Especially for regeneration of large bone defects, where the requirements for grafting material are substantial, these synthetics can be used in combination with autologous bone graft, growth factors or cells [8]. Furthermore, there are also non-biological osteoconductive substrates, such as fabricated biocompatible metals (for example, porous tantalum) that offer the potential for absolute control of the final structure without any immunogenicity [8].

Research is ongoing to improve the mechanical properties and biocompatibility of scaffolds, to promote osteoblast adhesion, growth and differentiation, and t0 allow vascular ingrowth and bone-tissue formation. Improved biodegradable and bioactive three-dimensional porous scaffolds [55] are being investigated, as well as novel approaches using nanotechnology, such as magnetic biohybrid porous scaffolds acting as a crosslinking agent for collagen for bone regeneration guided by an external magnetic field [56], or injectable scaffolds for easier application [57].

## Tissue engineering

The tissue-engineering approach is a promising strategy added in the field of bone regenerative medicine, which aims to generate new, cell-driven, functional tissues, rather than just to implant non-living scaffolds [58]. This alternative treatment of conditions requiring bone regeneration could overcome the limitations of current therapies, by combining the principles of orthopaedic surgery with knowledge from biology, physics, materials science and engineering, and its clinical application offers great potential [58,59]. In essence, bone-tissue engineering combines progenitor cells, such as MSCs (native or expanded) or mature cells (for osteogenesis) seeded in biocompatible scaffolds and ideally in three-dimensional tissue-like structures (for osteoconduction and vascular ingrowth), with appropriate growth factors (for osteoinduction), in order to generate and maintain bone [60]. The need for such improved composite grafts is obvious, especially for the management of large bone defects, for which the requirements for grafting material are substantial [8]. At present, composite grafts that are available include bone synthetic or bioabsorbable scaffolds seeded with bone-marrow aspirate or growth factors (BMPs), providing a competitive alternative to autologous bone graft [8].

Several major technical advances have been achieved in the field of bone-tissue engineering during the past decade, especially with the increased understanding of bone healing at the molecular and cellular level, allowing the conduction of numerous animal studies and of the first pilot clinical studies using tissue-engineered constructs for local bone regeneration. To date, seven human studies have been conducted using culture-expanded, non-genetically modified MSCs for regeneration of bone defects: two studies reporting on long bones and five on maxillofacial conditions [61]. Even though they are rather heterogeneous studies and it is difficult to draw conclusive evidence from them, bone apposition by the grafted MSCs was seen, but it was not sufficient to bridge large bone defects. Furthermore, the tissue-engineering approach has been used to accelerate the fracture-healing process or to augment the bone-prosthesis interface and prevent aseptic loosening in total joint arthroplasty, with promising results regarding its efficacy and safety [62,63].

Recently, an animal study has shown the potential for prefabrication of vascularised bioartificial bone grafts *in vivo *using β-TCP scaffolds intraoperatively filled with autogenous bone marrow for cell loading, and implanted into the latissimus dorsi muscle for potential application at a later stage for segmental bone reconstruction, introducing the principles of bone transplantation with minimal donor-site morbidity and no quantity restrictions [64].

Overall, bone-tissue engineering is in its infancy, and there are many issues of efficacy, safety and cost to be addressed before general clinical application can be achieved. Cultured-expanded cells may have mutations or epigenetic changes that could confer a tumour-forming potential [44]. However, *in vitro and in vivo *evidence suggests that the risk of tumour formation is minimal [65]. No cases of tumour transformation were reported in 41 patients (45 joints) after autologous bone-marrow-derived MSC transplantation for cartilage repair, who were followed for up to 11 years and 5 months [46]. Another important issue is the difficulty of achieving an effective and high-density cell population within three-dimensional biodegradable scaffolds [66]. Consequently, numerous bioreactor technologies have been investigated, and many others should be developed [67]. Their degradation properties should also preserve the health of local tissues and the continuous remodelling of bone [44]. Three-dimensional porous scaffolds with specific architectures at the nano, micro and macro scale for optimum surface porosity and chemistry, which enhance cellular attachment, migration, proliferation and differentiation, are undergoing a continuous evaluation process.

## Gene therapy

Another promising method of growth-factor delivery in the field of bone-tissue engineering is the application of gene therapy [68,69]. This involves the transfer of genetic material into the genome of the target cell, allowing expression of bioactive factors from the cells themselves for a prolonged time. Gene transfer can be performed using a viral (transfection) or a non-viral (transduction) vector, and by either an *in vivo or ex vivo *gene-transfer strategy. With the *in vivo *method, which is technically relatively easier, the genetic material is transferred directly into the host; however, there are safety concerns with this approach. The indirect *ex vivo *technique requires the collection of cells by tissue harvest, and their genetic modification *in vitro *before transfer back into the host. Although technically more demanding, it is a safer method, allowing testing of the cells for any abnormal behaviour before reimplantation, and selection of those with the highest gene expression [69].

Besides the issues of cost, efficacy and biological safety that need to be answered before this strategy of genetic manipulation is applied in humans, delivery of growth factors, particularly BMPs, using gene therapy for bone regeneration has already produced promising results in animal studies [70,71].

## Mechanical stability and the role of mechanical stimulation in bone regeneration

In addition to the intrinsic potential of bone to regenerate and to the aforementioned methods used to enhance bone regeneration, adequate mechanical stability by various means of stabilisation and use of fixation devices is also an important element for optimal bone repair, especially in challenging cases involving large bone defects or impaired bone healing. The mechanical environment constitutes the fourth factor of the 'diamond concept' of fracture healing, along with osteoconductive scaffolds, growth factors and osteogenic cells, interacting during the repair process [28].

During bone regeneration, intermediate tissues, such as fibrous connective tissue, cartilage and woven bone, precede final bone formation, providing initial mechanical stability and a scaffold for tissue differentiation. The mechanical loading affects the regeneration process, with different stress distribution favouring or inhibiting differentiation of particular tissue phenotypes [72]. High shear strain and fluid flows are thought to stimulate formation of fibrous connective tissue, whereas lower levels stimulate formation of cartilage, and even lower levels favour ossification [72].

The interfragmentary strain concept of Perren has been used to describe the different patterns of bone repair (primary or secondary fracture healing), suggesting that the strain that causes healthy bone to fail is the upper limit that can be tolerated for the regenerating tissue [73]. Since then, extensive research on this field has further refined the effects of mechanical stability and mechanical stimulation on bone regeneration and fracture healing [74]. Numerous *in vivo *studies have shown contradictory results regarding the contribution of strain and mechanical stimulation, in terms of compression or distraction, in bone formation during fracture healing. In early fracture healing, mechanical stimulation seems to enhance callus formation, but the amount of callus formation does not correspond to stiffness [74]. During the initial stages of bone healing, a less rigid mechanical environment resulted in a prolonged chondral bone regeneration phase, whereas the process of intramembranous ossification appeared to be independent of mechanical stability [75]. By contrast, a more rigid mechanical environment resulted in a smaller callus and a reduced fibrous-tissue component [76]. For later stages of bone regeneration, lower mechanical stability was found to inhibit callus bridging and stiffness [74]. Finally, *in vitro *studies have also shown the role of the mechanical environment on different cell types involved in bone regeneration. It has been demonstrated using cell-culture systems that the different cellular responses in terms of proliferation and differentiation after mechanical stimulation depend on the strain magnitude and the cell phenotype [74].

Mechanical stability is also important for local vascularisation and angiogenesis during bone regeneration. In an i*n vivo *study, it was shown that smaller interfragmentary movements led to the formation of a greater number of vessels within the callus, particularly in areas close to the periosteum, compared with larger movements [77], whereas increased interfragmentary shear was associated with reduced vascularisation with a higher amount of fibrous-tissue formation and a lower percentage of mineralised bone during early bone healing [78].

Finally, the presence of a mechanically stable environment throughout the bone-regeneration process is also essential when additional methods are being used to enhance bone repair [28,79]. Optimal instrumentation with minimal disruption of the local blood supply is required to supplement and protect the mechanical properties of the implanted grafts or scaffolds to allow incorporation, vascularisation and subsequent remodelling [79].

## Systemic enhancement of bone regeneration

As an alternative to local augmentation of the bone-regeneration process, the use of systemic agents, including growth hormone (GH) [80] and parathyroid hormone (PTH) [81] is also under extensive research. Current evidence suggests a positive role for GH in fracture healing, but there are issues about its safety profile and optimal dose, when systemically administered to enhance bone repair [80]. There are also numerous animal studies and clinical trials showing that intermittent PTH administration induces both cancellous and cortical bone regeneration, enhances bone mass, and increases mechanical bone strength and bone-mineral density, with a relatively satisfactory safety profile [81,82]. Currently, two PTH analogues, PTH 1-34 (or teriparitide) and PTH 1-84, are already used in clinical practice as anabolic agents for the treatment of osteoporosis [81,83], and research is being carried out into their off-label use as bone-forming agents in complex conditions requiring enhancement of bone repair, such as complicated fractures and non-unions.

In addition to the anabolic agents for bone regeneration, current antiresorptive therapies that are already in clinical use for the management of osteoporosis could be used to increase bone-mineral density during bone regeneration and remodelling by reducing bone resorption. Biphosphonates, known to reduce the recruitment and activity of osteoclasts and increase their apoptosis, and strontium ranelate, known to inhibit bone resorption and stimulate bone formation, could be beneficial adjuncts to bone repair by altering bone turnover [84]. In addition, a new pharmaceutical agent called denosumab, which is a fully human monoclonal antibody designed to target receptor activator of nuclear factor-κB ligand (RANKL), a protein that selectively inhibits osteoclastogenesis, might not only decrease bone turnover and increase bone-mineral density in osteoporosis, but also indirectly improve bone regeneration in other conditions requiring enhancement [85].

Recently, another signalling pathway, the Wnt pathway, was found to play a role in bone regeneration [86]. Impaired Wnt signalling is associated with osteogenic pathologies, such as osteoporosis and osteopenia. Thus, novel strategies that systemically induce the Wnt signalling pathway or inhibit its antagonists, such as sclerostin, can improve bone regeneration. However, there are concerns about carcinogenesis [87].

Another approach for systemic enhancement of bone regeneration is the use of agonists of the prostaglandin receptors EP2 and EP4, which were found to be skeletally anabolic at cortical and cancellous sites. Promising results have been seen in animal models, without adverse effects, and therefore these receptors may represent novel anabolic agents for the treatment of osteoporosis and for augmentation of bone healing [27].

Finally, new treatments for systemic augmentation of bone regeneration may come to light while researchers are trying to elucidate the alterations seen at the cellular and molecular level in conditions with increased bone formation capacity. Fibrodysplasia ossificans progressiva, a rare genetic disorder, is an example of how an abnormal metabolic condition can be viewed as evidence for systemic regeneration of large amounts of bone secondary to alterations within the BMP signalling pathway [88], and may indicate unique treatment potentials.

## Conclusions

There are several clinical conditions that require enhancement of bone regeneration either locally or systemically, and various methods are currently used to augment or accelerate bone repair, depending on the healing potential and the specific requirements of each case. Knowledge of bone biology has vastly expanded with the increased understanding at the molecular level, resulting in development of many new treatment methods, with many others (or improvements to current ones) anticipated in the years to come. However, there are still gaps; in particular, there is still surprisingly little information available about the cellular basis for MSC-mediated fracture repair and bone regeneration *in viv*o in humans. Further understanding in this area could be the key to an improved and integrated strategy for skeletal repair.

In the future, control of bone regeneration with strategies that mimic the normal cascade of bone formation will offer successful management of conditions requiring enhancement of bone regeneration, and reduce their morbidity and cost in the long term. Research is ongoing within all relevant fields, and it is hoped that many bone-disease processes secondary to trauma, bone resection due to ablative surgery, ageing, and metabolic or genetic skeletal disorders will be successfully treated with novel bone-regeneration protocols that may address both local and systemic enhancement to optimise outcome.

## Competing interests

The authors declare that they have no competing interests.

## Authors' contributions

RD contributed to the literature review and writing. EJ, DMcG and PVG contributed to the writing of specific sections of the manuscript within their main scientific interest, and critically revised the manuscript for important intellectual content. All authors read and have given final approval of the final manuscript.

## Pre-publication history

The pre-publication history for this paper can be accessed here:

http://www.biomedcentral.com/1741-7015/9/66/prepub

## References

[B1] Bates P Ramachandran M Ramachandran M Bone injury, healing and grafting Basic Orthopaedic Sciences. The Stanmore Guide 2007 London: Hodder Arnold 123 134

[B2] Einhorn TA The cell and molecular biology of fracture healing Clin Orthop Relat Res 1998 355 Suppl S7 21 9917622 10.1097/00003086-199810001-00003

[B3] Cho TJ Gerstenfeld LC Einhorn TA Differential temporal expression of members of the transforming growth factor beta superfamily during murine fracture healing J Bone Miner Res 2002 17 513 520 10.1359/jbmr.2002.17.3.513 11874242

[B4] Ferguson C Alpern E Miclau T Helms JA Does adult fracture repair recapitulate embryonic skeletal formation? Mech Dev 1999 87 57 66 10.1016/S0925-4773(99)00142-2 10495271

[B5] Audigé L Griffin D Bhandari M Kellam J Rüedi TP Path analysis of factors for delayed healing and nonunion in 416 operatively treated tibial shaft fractures Clin Orthop Relat Res 2005 438 221 232 16131895 10.1097/01.blo.0000163836.66906.74

[B6] Aronson J Limb-lengthening, skeletal reconstruction, and bone transport with the Ilizarov method J Bone Joint Surg Am 1997 79 8 1243 1258 9278087 10.2106/00004623-199708000-00019

[B7] Green SA Jackson JM Wall DM Marinow H Ishkanian J Management of segmental defects by the Ilizarov intercalary bone transport method Clin Orthop Relat Re 1992 280 136 142 1611733

[B8] Giannoudis PV Dinopoulos H Tsiridis E Bone substitutes: an update Injury 2005 36 Suppl 3 S20 27 16188545 10.1016/j.injury.2005.07.029

[B9] Giannoudis PV Einhorn TA Bone morphogenetic proteins in musculoskeletal medicine Injury 2009 40 Suppl 3 S1 3 20082783 10.1016/S0020-1383(09)00642-1

[B10] Masquelet AC Begue T The concept of induced membrane for reconstruction of long bone defects Orthop Clin North Am 2010 41 1 27 37 10.1016/j.ocl.2009.07.011 19931050

[B11] Busse JW Bhandari M Kulkarni AV Tunks E The effect of low-intensity pulsed ultrasound therapy on time to fracture healing: a meta-analysis CMAJ 2002 166 4 437 441 11873920 PMC99352

[B12] Schofer MD Block JE Aigner J Schmelz A Improved healing response in delayed unions of the tibia with low-intensity pulsed ultrasound: results of a randomized sham-controlled trial BMC Musculoskelet Disord 2010 11 229 10.1186/1471-2474-11-229 20932272 PMC2958986

[B13] Walker NA Denegar CR Preische J Low-intensity pulsed ultrasound and pulsed electromagnetic field in the treatment of tibial fractures: a systematic review J Athl Train 2007 42 4 530 535 18174942 PMC2140080

[B14] Raschke M Oedekoven G Ficke J Claudi BF The monorail method for segment bone transport Injury 1993 24 Suppl 2 S54 61 8188331 10.1016/0020-1383(93)90020-7

[B15] Cole JD Justin D Kasparis T DeVlught D Knobloch C The intramedullary skeletal kinetic distractor (ISKD): first clinical results of a new intramedullary nail for lengthening of the femur and tibia Injury 2001 32 Suppl 4 129 139 11812486 10.1016/s0020-1383(01)00116-4

[B16] Bauer TW Muschler GF Bone graft materials. An overview of the basic science Clin Orthop Relat Res 2000 371 10 27 10693546

[B17] Pederson WC Person DW Long bone reconstruction with vascularized bone grafts Orthop Clin North Am 2007 38 1 23 35 10.1016/j.ocl.2006.10.006 17145292

[B18] Korompilias AV Beris AE Lykissas MG Kostas-Agnantis IP Soucacos PN Femoral head osteonecrosis: Why choose free vascularized fibula grafting Microsurgery 2010 in press 10.1002/micr.20837 21400578

[B19] Giannoudis PV Tzioupis C Green J Surgical techniques: how I do it? The reamer/irrigator/aspirator (RIA) system Injury 2009 40 11 1231 1236 10.1016/j.injury.2009.07.070 19783249

[B20] Ahlmann E Patzakis M Roidis N Shepherd L Holtom P Comparison of anterior and posterior iliac crest bone graft in terms of harvest-site morbidity and functional outcomes J Bone Joint Surg Am 2002 84 5 716 720 10.1302/0301-620X.84B5.12571 12004011

[B21] St John TA Vaccaro AR Sah AP Schaefer M Berta SC Albert T Hilibrand A Physical and monetary costs associated with autogenous bone graft harvesting Am J Orthop 2003 32 1 18 23 12580346

[B22] Younger EM Chapman MW Morbidity at bone graft donor sites J Orthop Trauma 1989 3 3 192 195 10.1097/00005131-198909000-00002 2809818

[B23] Finkemeier CG Bone-grafting and bone-graft substitutes J Bone Joint Surg Am 2002 84 3 454 464 11886919 10.2106/00004623-200203000-00020

[B24] Bullens PH Bart Schreuder HW de Waal Malefijt MC Verdonschot N Buma P Is an impacted morselized graft in a cage an alternative for reconstructing segmental diaphyseal defects? Clin Orthop Relat Res 2009 467 3 783 791 10.1007/s11999-008-0686-5 19142693 PMC2635451

[B25] Ostermann PA Haase N Rübberdt A Wich M Ekkernkamp A Management of a long segmental defect at the proximal meta-diaphyseal junction of the tibia using a cylindrical titanium mesh cage J Orthop Trauma 2002 16 8 597 601 10.1097/00005131-200209000-00010 12352570

[B26] Urist MR O'Connor BT Burwell RG Bone Graft Derivatives and Substitutes 1994 Oxford: Butterworth-Heinemann Ltd

[B27] Komatsu DE Warden SJ The control of fracture healing and its therapeutic targeting: improving upon nature J Cell Biochem 2010 109 2 302 311 19950200 10.1002/jcb.22418

[B28] Giannoudis PV Einhorn TA Marsh D Fracture healing: the diamond concept Injury 2007 38 Suppl 4 S3 6 18224731 10.1016/s0020-1383(08)70003-2

[B29] Dimitriou R Tsiridis E Giannoudis PV Current concepts of molecular aspects of bone healing Injury 2005 36 12 1392 1404 10.1016/j.injury.2005.07.019 16102764

[B30] Food and Drug Administration Medical devices. [http://www.fda.gov/MedicalDevices/ProductsandMedicalProcedures/DeviceApprovalsandClearances/Recently-ApprovedDevices/default.htm]

[B31] Blokhuis TJ Formulations and delivery vehicles for bone morphogenetic proteins: latest advances and future directions Injury 2009 40 Suppl 3 S8 11 20082796 10.1016/S0020-1383(09)70004-X

[B32] Nauth A Giannoudis PV Einhorn TA Hankenson KD Friedlaender GE Li R Schemitsch EH Growth factors: beyond bone morphogenetic proteins J Orthop Trauma 2010 24 9 543 546 10.1097/BOT.0b013e3181ec4833 20736791

[B33] Simpson AH Mills L Noble B The role of growth factors and related agents in accelerating fracture healing J Bone Joint Surg Br 2006 88 6 701 705 10.1302/0301-620X.88B6.17524 16720758

[B34] Alsousou J Thompson M Hulley P Noble A Willett K The biology of platelet-rich plasma and its application in trauma and orthopaedic surgery: a review of the literature J Bone Joint Surg Br 2009 91 8 987 996 10.1302/0301-620X.91B8.22546 19651823

[B35] Argintar E Edwards S Delahay J Bone morphogenetic proteins in orthopaedic trauma surgery Injury 2010 in press 10.1016/j.injury.2010.11.016 21145058

[B36] Chen FM Ma ZW Dong GY Wu ZF Composite glycidyl methacrylated dextran (Dex-GMA)/gelatin nanoparticles for localized protein delivery Acta Pharmacol Sin 2009 30 4 485 493 10.1038/aps.2009.15 19305420 PMC4002268

[B37] Pountos I Georgouli T Kontakis G Giannoudis PV Efficacy of minimally invasive techniques for enhancement of fracture healing: evidence today Int Orthop 2010 34 1 3 12 10.1007/s00264-009-0892-0 19844709 PMC2899260

[B38] D'Ippolito G Schiller PC Ricordi C Roos BA Howard GA Age-related osteogenic potential of mesenchymal stromal stem cells from human vertebral bone marrow J Bone Miner Res 1999 14 7 1115 1122 10.1359/jbmr.1999.14.7.1115 10404011

[B39] Huibregtse BA Johnstone B Goldberg VM Caplan AI Effect of age and sampling site on the chondro-osteogenic potential of rabbit marrow-derived mesenchymal progenitor cells J Orthop Res 2000 18 1 18 24 10.1002/jor.1100180104 10716274

[B40] Hernigou P Poignard A Beaujean F Rouard H Percutaneous autologous bone-marrow grafting for nonunions. Influence of the number and concentration of progenitor cells J Bone Joint Surg Am 2005 87 7 1430 1437 10.2106/JBJS.D.02215 15995108

[B41] Jäger M Herten M Fochtmann U Fischer J Hernigou P Zilkens C Hendrich C Krauspe R Bridging the gap: bone marrow aspiration concentrate reduces autologous bone grafting in osseous defects J Orthop Res 2011 29 2 173 180 10.1002/jor.21230 20740672

[B42] Bianchi G Banfi A Mastrogiacomo M Notaro R Luzzatto L Cancedda R Quarto R Ex vivo enrichment of mesenchymal cell progenitors by fibroblast growth factor 2 Exp Cell Res 2003 287 1 98 105 10.1016/S0014-4827(03)00138-1 12799186

[B43] D'Ippolito G Diabira S Howard GA Menei P Roos BA Schiller PC Marrow-isolated adult multilineage inducible (MIAMI) cells, a unique population of postnatal young and old human cells with extensive expansion and differentiation potential J Cell Sci 2004 117 14 2971 2981 10.1242/jcs.01103 15173316

[B44] Patterson TE Kumagai K Griffith L Muschler GF Cellular strategies for enhancement of fracture repair J Bone Joint Surg Am 2008 90 Suppl 1 111 119 18292365 10.2106/JBJS.G.01572

[B45] McGonagle D English A Jones EA The relevance of mesenchymal stem cells in vivo for future orthopaedic strategies aimed at fracture repair Curr Orthop 2007 21 4 262 267 10.1016/j.cuor.2007.07.004

[B46] Wakitani S Okabe T Horibe S Mitsuoka T Saito M Koyama T Nawata M Tensho K Kato H Uematsu K Kuroda R Kurosaka M Yoshiya S Hattori K Ohgushi H Safety of autologous bone marrow-derived mesenchymal stem cell transplantation for cartilage repair in 41 patients with 45 joints followed for up to 11 years and 5 months J Tissue Eng Regen Med 2011 5 2 146 150 10.1002/term.299 20603892

[B47] Matsumoto T Kawamoto A Kuroda R Ishikawa M Mifune Y Iwasaki H Miwa M Horii M Hayashi S Oyamada A Nishimura H Murasawa S Doita M Kurosaka M Asahara T Therapeutic potential of vasculogenesis and osteogenesis promoted by peripheral blood CD34-positive cells for functional bone healing Am J Pathol 2006 169 1440 1457 10.2353/ajpath.2006.060064 17003498 PMC1698844

[B48] Zuk PA Zhu M Mizuno H Huang J Futrell JW Katz AJ Benhaim P Lorenz HP Hedrick MH Multilineage cells from human adipose tissue: implications for cell-based therapies Tissue Eng 2001 7 2 211 228 10.1089/107632701300062859 11304456

[B49] Jackson WM Aragon AB Djouad F Song Y Koehler SM Nesti LJ Tuan RS Mesenchymal progenitor cells derived from traumatized human muscle J Tissue Eng Regen Med 2009 3 2 129 138 10.1002/term.149 19170141 PMC2814161

[B50] Im GI Shin YW Lee KB Do adipose tissue-derived mesenchymal stem cells have the same osteogenic and chondrogenic potential as bone marrow-derived cells? Osteoarthritis Cartilage 2005 13 10 845 853 10.1016/j.joca.2005.05.005 16129630

[B51] Niemeyer P Fechner K Milz S Richter W Suedkamp NP Mehlhorn AT Pearce S Kasten P Comparison of mesenchymal stem cells from bone marrow and adipose tissue for bone regeneration in a critical size defect of the sheep tibia and the influence of platelet-rich plasma Biomaterials 2010 31 13 3572 3529 10.1016/j.biomaterials.2010.01.085 20153047

[B52] Jones E McGonagle D Human bone marrow mesenchymal stem cells in vivo Rheumatology (Oxford) 2008 47 2 126 131 10.1093/rheumatology/kem206 17986482

[B53] Jones EA Kinsey SE English A Jones RA Straszynski L Meredith DM Markham AF Jack A Emery P McGonagle D Isolation and characterization of bone marrow multipotential mesenchymal progenitor cells Arthritis Rheum 2002 46 12 3349 3360 10.1002/art.10696 12483742

[B54] Jones E English A Churchman SM Kouroupis D Boxall SA Kinsey S Giannoudis PG Emery P McGonagle D Large-scale extraction and characterization of CD271+ multipotential stromal cells from trabecular bone in health and osteoarthritis: implications for bone regeneration strategies based on uncultured or minimally cultured multipotential stromal cells Arthritis Rheum 2010 62 7 1944 1954 20222109 10.1002/art.27451

[B55] Akkouch A Zhang Z Rouabhia M A novel collagen/hydroxyapatite/poly(lactide-co-ε-caprolactone) biodegradable and bioactive 3D porous scaffold for bone regeneration J Biomed Mater Res A 2011 96A 693 704 10.1002/jbm.a.33033 21284080

[B56] Tampieri A Landi E Valentini F Sandri M D'Alessandro T Dediu V Marcacci M A conceptually new type of bio-hybrid scaffold for bone regeneration Nanotechnology 2011 22 1 015104 10.1088/0957-4484/22/1/015104 21135464

[B57] Laschke MW Witt K Pohlemann T Menger MD Injectable nanocrystalline hydroxyapatite paste for bone substitution: in vivo analysis of biocompatibility and vascularization J Biomed Mater Res B Appl Biomater 2007 82 2 494 505 17279565 10.1002/jbm.b.30755

[B58] Salgado AJ Coutinho OP Reis RL Bone tissue engineering: state of the art and future trends Macromol Biosci 2004 4 8 743 765 10.1002/mabi.200400026 15468269

[B59] Rose FR Oreffo RO Bone tissue engineering: hope vs hype Biochem Biophys Res Commun 2002 292 1 7 10.1006/bbrc.2002.6519 11890663

[B60] Jones EA Yang XB Mesenchymal stem cells and their future in bone repair Int J Adv Rheumatol 2005 3 3 15 21

[B61] Chatterjea A Meijer G van Blitterswijk C de Boer J Clinical application of human mesenchymal stromal cells for bone tissue engineering Stem Cells Int 2010 2010 215625 21113294 10.4061/2010/215625 PMC2989379

[B62] Kim SJ Shin YW Yang KH Kim SB Yoo MJ Han SK Im SA Won YD Sung YB Jeon TS Chang CH Jang JD Lee SB Kim HC Lee SY A multi-center, randomized, clinical study to compare the effect and safety of autologous cultured osteoblast (Ossron) injection to treat fractures BMC Musculoskelet Disord 2009 10 20 10.1186/1471-2474-10-20 19216734 PMC2656455

[B63] Ohgushi H Kotobuki N Funaoka H Machida H Hirose M Tanaka Y Takakura Y Tissue engineered ceramic artificial joint--ex vivo osteogenic differentiation of patient mesenchymal cells on total ankle joints for treatment of osteoarthritis Biomaterials 2005 26 22 4654 4661 10.1016/j.biomaterials.2004.11.055 15722135

[B64] Kokemueller H Spalthoff S Nolff M Tavassol F Essig H Stuehmer C Bormann KH Rücker M Gellrich NC Prefabrication of vascularized bioartificial bone grafts in vivo for segmental mandibular reconstruction: experimental pilot study in sheep and first clinical application Int J Oral Maxillofac Surg 2010 39 4 379 387 10.1016/j.ijom.2010.01.010 20167453

[B65] Tarte K Gaillard J Lataillade JJ Fouillard L Becker M Mossafa H Tchirkov A Rouard H Henry C Splingard M Dulong J Monnier D Gourmelon P Gorin NC Sensebé L Société Française de Greffe de Moelle et Thérapie Cellulaire Clinical-grade production of human mesenchymal stromal cells: occurrence of aneuploidy without transformation Blood 2010 115 8 1549 1553 10.1182/blood-2009-05-219907 20032501

[B66] Weinand C Xu JW Peretti GM Bonassar LJ Gill TJ Conditions affecting cell seeding onto three-dimensional scaffolds for cellular-based biodegradable implants J Biomed Mater Res B Appl Biomater 2009 91 1 80 87 19388093 10.1002/jbm.b.31376

[B67] Yoshioka T Mishima H Ohyabu Y Sakai S Akaogi H Ishii T Kojima H Tanaka J Ochiai N Uemura T Repair of large osteochondral defects with allogeneic cartilaginous aggregates formed from bone marrow-derived cells using RWV bioreactor J Orthop Res 2007 25 10 1291 1298 10.1002/jor.20426 17549704

[B68] Caplan AI Mesenchymal stem cells and gene therapy Clin Orthop Relat Res 2000 379 Suppl S67 70 11039754 10.1097/00003086-200010001-00010

[B69] Chen Y Orthopaedic application of gene therapy J Orthop Sci 2001 6 199 207 10.1007/s007760100072 11484110

[B70] Calori GM Donati D Di Bella C Tagliabue L Bone morphogenetic proteins and tissue engineering: future directions Injury 2009 40 Suppl 3 S67 76 20082795 10.1016/S0020-1383(09)70015-4

[B71] Tang Y Tang W Lin Y Long J Wang H Liu L Tian W Combination of bone tissue engineering and BMP-2 gene transfection promotes bone healing in osteoporotic rats Cell Biol Int 2008 32 9 1150 1157 10.1016/j.cellbi.2008.06.005 18638562

[B72] Lacroix D Prendergast PJ A mechano-regulation model for tissue differentiation during fracture healing: analysis of gap size and loading J Biomech 2002 35 9 1163 1171 10.1016/S0021-9290(02)00086-6 12163306

[B73] Perren SM Physical and biological aspects of fracture healing with special reference to internal fixation Clin Orthop Relat Res 1979 138 175 196 376198

[B74] Jagodzinski M Krettek C Effect of mechanical stability on fracture healing--an update Injury 2007 38 Suppl1 S3 10 17383483 10.1016/j.injury.2007.02.005

[B75] Epari DR Schell H Bail HJ Duda GN Instability prolongs the chondral phase during bone healing in sheep Bone 2006 38 6 864 870 10.1016/j.bone.2005.10.023 16359937

[B76] Schell H Epari DR Kassi JP Bragulla H Bail HJ Duda GN The course of bone healing is influenced by the initial shear fixation stability J Orthop Res 2005 23 5 1022 1028 10.1016/j.orthres.2005.03.005 15878254

[B77] Claes L Eckert-Hübner K Augat P The effect of mechanical stability on local vascularization and tissue differentiation in callus healing J Orthop Res 2002 20 5 1099 1105 10.1016/S0736-0266(02)00044-X 12382978

[B78] Lienau J Schell H Duda GN Seebeck P Muchow S Bail HJ Initial vascularization and tissue differentiation are influenced by fixation stability J Orthop Res 2005 23 3 639 645 10.1016/j.orthres.2004.09.006 15885486

[B79] Babis GC Soucacos PN Bone scaffolds: The role of mechanical stability and instrumentation Injury 2005 36 Suppl S38 S44 16291322 10.1016/j.injury.2005.10.009

[B80] Tran GT Pagkalos J Tsiridis E Narvani AA Heliotis M Mantalaris A Tsiridis E Growth hormone: does it have a therapeutic role in fracture healing? Expert Opin Investig Drugs 2009 18 7 887 911 10.1517/13543780902893069 19480608

[B81] Rubin MR Bilezikian JP Parathyroid hormone as an anabolic skeletal therapy Drugs 2005 65 17 2481 2498 10.2165/00003495-200565170-00005 16296873

[B82] Tzioupis CC Giannoudis PV The safety and efficacy of parathyroid hormone (PTH) as a biological response modifier for the enhancement of bone regeneration Curr Drug Saf 2006 1 2 189 203 10.2174/157488606776930571 18690930

[B83] Verhaar HJ Lems WF PTH analogues and osteoporotic fractures Expert Opin Biol Ther 2010 10 9 1387 1394 10.1517/14712598.2010.506870 20629581

[B84] Kanis JA Burlet N Cooper C Delmas PD Reginster JY Borgstrom F Rizzoli R European Society for Clinical and Economic Aspects of Osteoporosis and Osteoarthritis (ESCEO): European guidance for the diagnosis and management of osteoporosis in postmenopausal women Osteoporos Int 2008 19 4 399 428 10.1007/s00198-008-0560-z 18266020 PMC2613968

[B85] Charopoulos I Orme S Giannoudis PV The role and efficacy of denosumab in the treatment of osteoporosis: an update Expert Opin Drug Saf 2011 in press 10.1517/14740338.2010.516249 21208140

[B86] Chen Y Alman BA Wnt pathway, an essential role in bone regeneration J Cell Biochem 2009 106 3 353 362 10.1002/jcb.22020 19127541

[B87] Wagner ER Zhu G Zhang BQ Luo Q Shi Q Huang E Gao Y Gao JL Kim SH Rastegar F Yang K He BC Chen L Zuo GW Bi Y Su Y Luo J Luo X Huang J Deng ZL Reid RR Luu HH Haydon RC He TC The therapeutic potential of the Wnt signaling pathway in bone disorders Curr Mol Pharmacol 2011 4 1 14 25 10.2174/1874467211104010014 20825362

[B88] Lucotte G Houzet A Hubans C Lagarde JP Lenoir G Mutations of the noggin (NOG) and of the activin A type I receptor (ACVR1) genes in a series of twenty-seven French fibrodysplasia ossificans progressiva (FOP) patients Genet Couns 2009 20 1 53 62 19400542

